# Rabex-5 E3 and Rab5 GEF domains differ in their regulation of Ras, Notch, and PI3K signaling in *Drosophila* wing development

**DOI:** 10.1371/journal.pone.0312274

**Published:** 2024-10-28

**Authors:** Theresa A. Reimels, Mia Steinberg, Hua Yan, Sivan Shahar, Ashley Rosenberg, Kristina Kalafsky, Max Luf, Lindsay Kelly, Stacia Octaviani, Cathie M. Pfleger

**Affiliations:** 1 Department of Oncological Sciences, Salt Lake City, Utah, United States of America; 2 The Graduate School of Biomedical Sciences, The Icahn School of Medicine at Mount Sinai, New York, New York, United States of America; 3 Department of Biology, University of Florida, Gainesville, Florida, United States of America; 4 The Tisch Cancer Institute, The Icahn School of Medicine at Mount Sinai, New York, NY, United States of America; Baylor College of Medicine, UNITED STATES OF AMERICA

## Abstract

Rabex-5 (also called RabGEF1), a protein originally characterized for its Rab5 GEF function, also has an A20-like E3 ubiquitin ligase domain. We and others reported that Rabex-5 E3 activity promotes Ras mono- and di-ubiquitination to inhibit Ras signaling in *Drosophila* and mammals. Subsequently, we reported that Rabex-5 inhibits Notch signaling in the *Drosophila* hematopoietic system. Here we report genetic interactions using Rabex-5 transgenes encoding domain-specific mutations that show that Rabex-5 requires an intact E3 domain to inhibit Notch signaling in the epithelial tissue of the developing wing. Surprisingly, we discovered that Rabex-5 with an impaired E3 domain but active Rab5 GEF domain suppresses Notch loss-of-function phenotypes and enhances both Notch duplication phenotypes and activated Ras phenotypes consistent with a model that the Rab5 GEF activity of Rabex-5 might positively regulate Ras and Notch. Positive and negative regulation of developmental signaling by its different catalytic domains could allow Rabex-5 to precisely coordinate developmental signaling to fine-tune patterning. Finally, we report that Rabex-5 also inhibits the overgrowth due to loss of PTEN or activation of PI3K but not activation of AKT. Inhibition of Ras, Notch, and PI3K signaling may explain why Rabex-5 is deleted in some cancers. Paradoxically, Rabex-5 is reported to be an oncogene in other cancers. We propose that Rabex-5 acts as a tumor suppressor via its E3 activity to inhibit Ras, Notch, and PI3K signaling and as an oncogene via its Rab5 GEF activity to enhance Ras and Notch signaling.

## Introduction

A number of signaling pathways play important roles in developmental biology and disease by regulating proliferation, cell growth, cell survival, cell fate, developmental patterning, and other key processes. Key components of these pathways can be regulated by different mechanisms including by post-translational modifications and by trafficking to change their localization within the cell. Rabex-5 (also called RabGEF1) was originally identified based on its ability to act as a GEF for endocytosis regulator Rab5 [1]. Rabex-5 has a second catalytic domain–an A20-like E3 ubiquitin protein ligase domain–which inhibits Ras signaling in both flies and mammals via its E3 domain by promoting the mono- and di-ubiquitination of Ras itself [2–6]. Ras regulates proliferation, cell survival, and cell fate to play important roles in development and is often amplified or mutationally activated in cancer. Activated Ras can signal from the plasma membrane and the Golgi; Rabex-5-promoted Ras mono- and di-ubiquitination sequesters Ras away from the Golgi and plasma membrane thereby preventing Ras from signaling to downstream effector ERK [2,6]. In *Drosophila*, inhibition of Ras by Rabex-5 was demonstrated in the epithelial tissues of the eye and the wing [4,6].

Larvae null for Rabex-5 demonstrated Notch-dependent hematopoietic phenotypes leading to the observation that Rabex-5 inhibits Notch signaling in *Drosophila* hematopoiesis in a Ras-independent manner [7]. This raised multiple questions including (1) if Rabex-5 regulation of Notch extends beyond the hematopoietic system to epithelial tissues, (2) if Rabex-5 inhibits Notch signaling via its E3 domain akin to its inhibition of Ras or via another domain such as the Rab5 GEF domain, and (3) if Rabex-5 regulates other important developmental signaling cascades.

We report here that Rabex-5 can also regulate Notch and Phosphatidylinositol 3-kinase (PI3K) in the wing epithelium concurrent to its regulation of Ras. Genetic interactions using Rabex-5 domain-specific mutant transgenes are consistent with a model that in addition to inhibiting Ras, Rabex-5 E3 activity can inhibit Notch signaling and PI3K signaling. Surprisingly, our genetic interaction data are consistent with a model that Rabex-5 Rab5 GEF activity can enhance both Ras and Notch signaling.

## Results and discussion

### Rabex-5 negatively regulates Notch signaling in the wing

**Fig 1:**
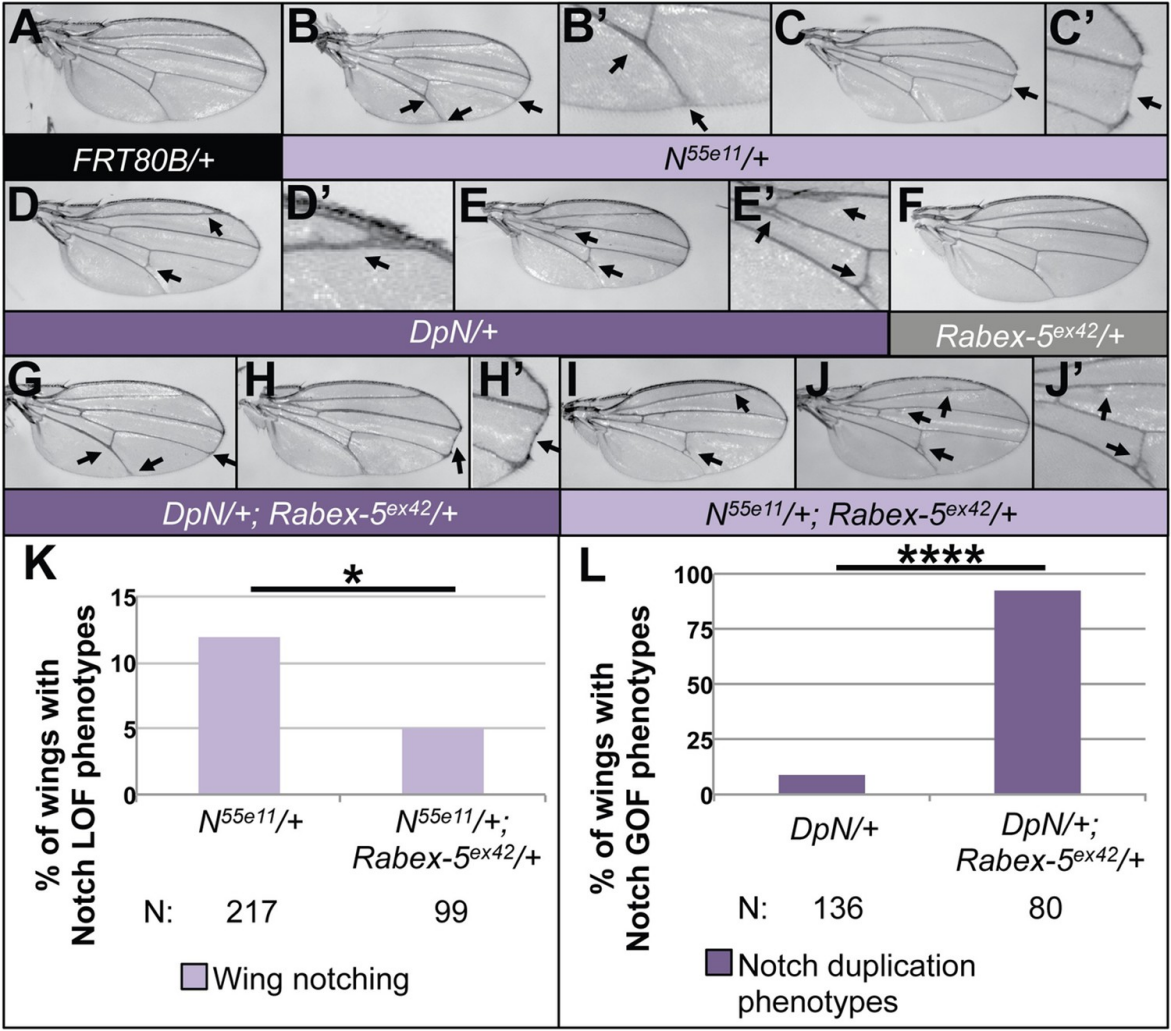
Rabex-5 interacts with Notch in the wing. (A) Control *FRT80B/+* wing (genotype *w*^*1118*^*; FRT80B/+*). (B-C’) Examples showing (B) effects on vein thickness or veins at the wing margin (arrows, enlarged in B’) and (C) the classic “notching” phenotype (arrow, enlarged in C’) upon heterozygosity for *Notch* loss-of-function allele *N*^*55e11*^ (genotype *N*^*55e11*^*/w*^*1118*^*; FRT80B/+*). (D-E’) Examples showing typical DpN vein effects (arrows) including extra wing vein material at L2 (D, enlarged in D’), the posterior crossvein (PCV E, enlarged in E’), and L3 (E, enlarged in E’) (genotype *w*^*1118*^; *DpN/+; FRT80B/+*). (F) Wing heterozygous for Rabex-5 null allele Rabex-5^ex42^ showing no visible phenotypes (genotype *w*^*1118*^*; FRT80B Rabex-5*^*ex42*^*/+*). (G-H’) Examples showing genetic interactions between *Rabex*-5^ex42^ and *N*^*55e11*^ (genotype *N*^*55e11*^*/w*^*1118*^*; FRT80B Rabex-5*^*ex42*^*/+*) showcasing wings with veination changes (G) or the classical wing notching (H, enlarged in H’). There is a decreased frequency of wing notching (quantified later in K), but there are still wings with the classical *Notch* loss-of-function phenotypes resembling wings in B-C’. (I-J’) Examples showing genetic interactions between *DpN* and Rabex-5^ex42^ (genotype *w*^*1118*^; *DpN/+; FRT80B Rabex-5*^*ex42*^*/+*) showcasing some of the ectopic wing veins and effects on the PCV (which resemble DpN wing phenotypes shown in D-E’) with an enlarged view of the wing in J shown in J’. The number of wings showing *Notch* duplication phenotypes increases (quantified later in L). (K) Graph summarizing genetic interactions between *Rabex*-5^ex42^ and *N*^*55e11*^. *Rabex*-5^ex42^ suppresses the *N*^*55e11*^ phenotypes. * indicates p = 0.0273 in chi square statistical tests. (L) Graph summarizing interactions between *DpN* and Rabex-5^ex42^. *Rabex*-5^ex42^ enhances *DpN* phenotypes. **** indicates p<0.0001 in chi square statistical tests. Number of flies (N) for each genotype is indicated below graphs in G-H and for graphs in Figs 2 and 3. Female wings are shown in this and subsequent figures. P values for this and subsequent figures are listed in S1 File.

To determine if Rabex-5 inhibition of Notch extended to tissues outside the hematopoietic system [7], we performed genetic interactions between Notch and Rabex-5 in the wing. Wings of flies heterozygous for loss-of-function allele *N*^*55e11*^ [8] have thickened veins, disrupted veins and bristles at the wing margin, and the classical “notching” phenotype (examples, Fig 1B–1C’) compared to control wings (Fig 1A). In contrast, wings heterozygous for a Notch duplication (DpN) show distinct phenotypes including wing vein effects and ectopic wing veins typically at the posterior crossvein (PCV) and longitudinal veins (examples, Fig 1D–1E’). Removing one copy of Rabex-5 by introducing null allele *Rabex-5*^*ex42*^ has no dominant visible wing phenotype (Fig 1F) but decreased the frequency of wing notching of *N*^*55e11*^ by more than half (wing images shown in Fig 1G–1H’, quantified in Fig 1K) and increased the proportion of DpN phenotypes more than ten-fold (wing images shown in Fig 1I–1J’ quantified in Fig 1L). We cannot make conclusions about effects on the severity of phenotypes in these experiments due to the low number of wings exhibiting phenotypes for specific genotypes. However, the quantification shows obvious effects on the frequency of phenotypes across the population of flies of each genotype. These data are consistent with a normal role for Rabex-5 to inhibit Notch signaling in the wing.

### Rabex-5 transgenes encoding domain-specific mutations show opposing effects on Notch pathway phenotypes

**Fig 2:**
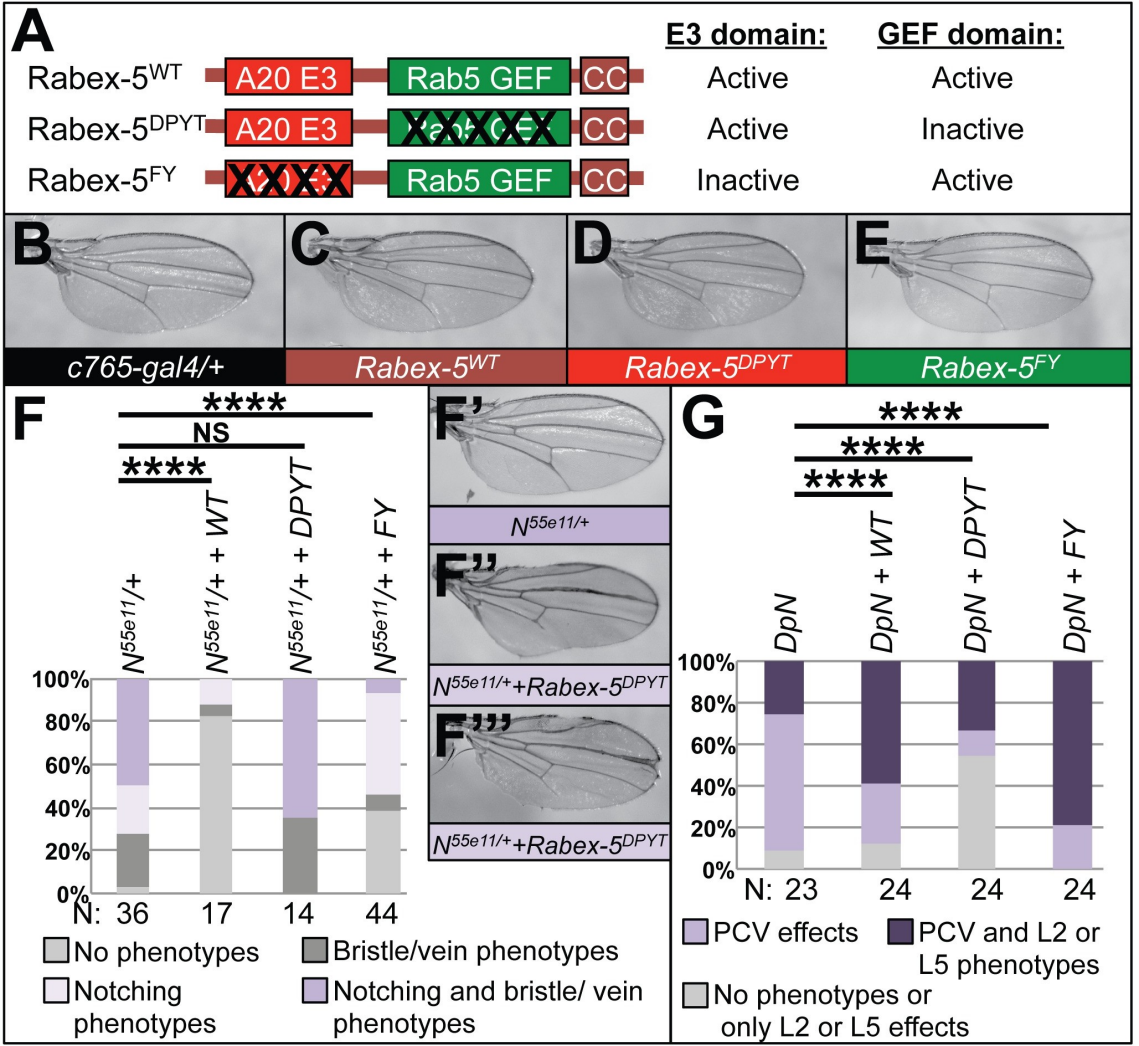
Rabex-5^DPYT^ and Rabex-5^FY^ show opposite effects on Notch signaling phenotypes. (A) Schematic summarizing the Rabex-5 catalytic domains and domain-specific transgenes used in this and subsequent figures. Rabex-5^DPYT^ encodes mutations inactivating Rab5 GEF activity but preserves E3 activity; Rabex-5^FY^ encodes mutations inactivating E3 activity but preserves Rab5 GEF activity. (B) Control *c765-gal4/+* wing (genotype *w*^*1118*^*; c765-gal4/+*). (C) Wing expressing Rabex-5^WT^ with *c765-gal4* (genotype *w*^*1118*^*; UAS Rabex-5*^*WT*^*/+; c765-gal4/+*). (D) Wing expressing Rabex-5^DPYT^ with *c765-gal4* (genotype *w*^*1118*^*; UAS Rabex-5*^*DPYT*^*/+; c765-gal4/+*). (E) Wing expressing Rabex-5^FY^ with *c765-gal4* (genotype *w*^*1118*^*; UAS Rabex-5*^*FY*^*/+; c765-gal4/+*). (F) Graph summarizing effects on *N*^*55e11*^ phenotypes (genotype *N*^*55e11*^*/w*^*1118*^*; c765-gal4/+*) when co-expressing Rabex-5^WT^, Rabex-5^DPYT^, or Rabex-5^FY^ with *c765-gal4* (genotypes *N*^*55e11*^*/w*^*1118*^*; UAS Rabex-5*^*WT*^*/+; c765-gal4/+*, *N*^*55e11*^*/w*^*1118*^*; UAS Rabex-5*^*DPYT*^*/+; c765-gal4/+*, and *N*^*55e11*^*/w*^*1118*^*; UAS Rabex-5*^*FY*^*/+; c765-gal4/+*, respectively). NS, P = 0.19, **** P<0.0001 in chi square statistical tests. Rabex-5^DPYT^ visibly enhances (although quantification of percent of phenotypes is not statistically significant) whereas Rabex-5^WT^ and Rabex-5^FY^ visibly and statistically suppress *N*^*55e11*^ phenotypes. (F’) *N*^*55e11*^*/+* control (genotype *N*^*55e11*^*/w*^*1118*^*; c765-gal4/+*). (F” and F”‘) *N*^*55e11*^*/+* wings expressing Rabex-5^DPYT^ using *c765-gal4* show the visibly enhanced phenotypes (genotype *N*^*55e11*^*/w*^*1118*^*; UAS Rabex-5*^*DPYT*^*/+; c765-gal4/+*). (G) Graph summarizing DpN (genotype *w*^*1118*^*; DpN/+; c765-gal4/+*) effects when co-expressing Rabex-5^WT^, Rabex-5^DPYT^, or Rabex-5^FY^ (genotypes *w*^*1118*^*; UAS Rabex-5*^*WT*^*/DpN; c765-gal4/+*, *w*^*1118*^*; UAS Rabex-5*^*DPYT*^*/DpN; c765-gal4/+*, and *w*^*1118*^*; UAS Rabex-5*^*FY*^*/DpN; c765-gal4/+*, respectively). ****indicates p<0.0001 in chi square statistical tests. Rabex-5^DPYT^ suppresses whereas Rabex-5^WT^ and Rabex-5^FY^ enhance *DpN* phenotypes.

To determine which domain of Rabex-5 underlies the interactions with Notch, we utilized transgenes we described previously [4] to express wild-type Rabex-5 and also transgenes encoding domain-specific mutations that inactivate catalytic functions (schematic, Fig 2A). Rabex-5^WT^ encodes wild-type Rabex-5 sequence. Rabex-5^DPYT^ contains alanine substitutions in D13, P320, Y357, and T360 to inactivate Rab5 GEF function without affecting E3 activity. Rabex-5^FY^ encodes alanine substitution of F25 and Y26 to inactivate E3 activity but preserve Rab5 GEF activity. Previous characterization [4] showed that these transgenes generally express to similar levels, although in some cases Rabex-5^DPYT^ showed decreased levels, presumably due to auto-ubiquitination activity (commonly seen for E3s). *N*^*55e11*^ and DpN exhibit different baseline phenotypes in different genetic backgrounds including in the presence of different Gal4 drivers. *c765-gal4* is primarily used as a wing driver due to its expression pattern across the entire wing [9,10], but it also has expression in other tissues including generalized expression in the thorax [11,12], in leg discs [13], and in the brain [14]. We see greater penetrance of *N*^*55e11*^ and *DpN* phenotypes in the presence of *c765-gal4* (compare left-most columns in 2F and 2G with the left columns in 1G and 1H, respectively). The increased baseline of phenotypes with *c765-gal4* creates a useful context to assess both enhancement and suppression of wing phenotypes.

As we showed previously, Rabex-5 transgene expression can cause wing phenotypes such as wing vein loss at higher levels of expression such as when using *engrailed-gal4* (*en-gal4*) [4]. *En-gal4* is useful as a wing driver because its expression pattern covers the entire posterior compartment, including strong expression in the posterior wing [15,16]. To avoid complications of interpretation, we established that the Rabex-5 transgenes expressed using *c765-gal4* at 21°C and 25°C did not result in visible phenotypes (Fig 2C–2E) such as wing vein loss, wing notching, or changes in wing area compared to controls (Fig 2B) unlike expression with *en-gal4* [4]. Consistent with Rabex-5 inhibition of Notch [7], expressing Rabex-5^DPYT^ visibly enhanced the severity of wing notching caused by *N*^*55e11*^ in the *c765-gal4* background (quantified in Fig 2F, wing examples, Fig 2F’-2F”‘). Given the lack of phenotype in control *c765>Rabex-5*^*DPYT*^ wings (Fig 2D), this increased severity can be interpreted as phenotypic enhancement. It is unclear if this enhancement results from further reduction in Notch transcriptional targets involved in wing development or engagement of other processes such as cell death in the context of a Notch loss-of-function. Consistent with the visible enhancement, there was a trend of increased percentage of wings with obvious Notch phenotypes (100% for co-expressing Rabex-5^DPYT^) reproducibly over several experiments. This was not always statistically significant, presumably in part because of the increased lethality of the genotype resulting in a decreased number of wings scored. Surprisingly, expressing Rabex-5^WT^ or Rabex-5^FY^ statistically significantly suppressed the percentage of wings with *N*^*55e11*^ phenotypes (quantified in Fig 2F). In interactions with DpN, Rabex-5^DPYT^ suppressed the DpN wing phenotypes, whereas Rabex-5^WT^ or Rabex-5^FY^ enhanced the DpN phenotypes (quantified in Fig 2G).

Our previous work suggested that Rabex-5 inhibits Notch in the hematopoietic system [7], and data in Fig 1 indicates that this role extends to the developing wing. The ability of Rabex-5^DPYT^ to suppress *N*^*55e11*^ phenotypes and enhance DpN phenotypes suggests that Rabex-5 Rab5 GEF activity is not required for Rabex-5 to inhibit Notch signaling. These interactions instead are consistent with a model that Rabex-5 E3 activity inhibits Notch signaling. Although we cannot rule out other interpretations (such as contributions from other sequence elements in Rabex-5), interactions between Rabex-5^FY^ and *N*^*55e11*^ and DpN are consistent with a model that Rabex-5 Rab5 GEF activity promotes or amplifies Notch signaling. This model is supported by other work showing that altered trafficking of Notch through the endosome can amplify Notch signaling or lead to ligand-independent Notch signaling [17–26]. It is perplexing that studies using null allele Rabex-5^ex42^ in Fig 1 could be interpreted as Rabex-5 inhibitory activity towards Notch, whereas over-expression studies using Rabex-5^WT^ in Fig 2 could be interpreted as Notch-promoting activity. We speculate that the balance of the opposing activities of the two catalytic domains depends on context and/or on the overall level of Rabex-5. We further speculate that in some contexts, or as the levels of Rabex-5 increase, the balance of activities favors the Rab5 GEF function to promote or amplify Notch signaling.

### Rabex-5 transgenes encoding domain-specific mutations show opposing effects on Ras pathway phenotypes

**Fig 3:**
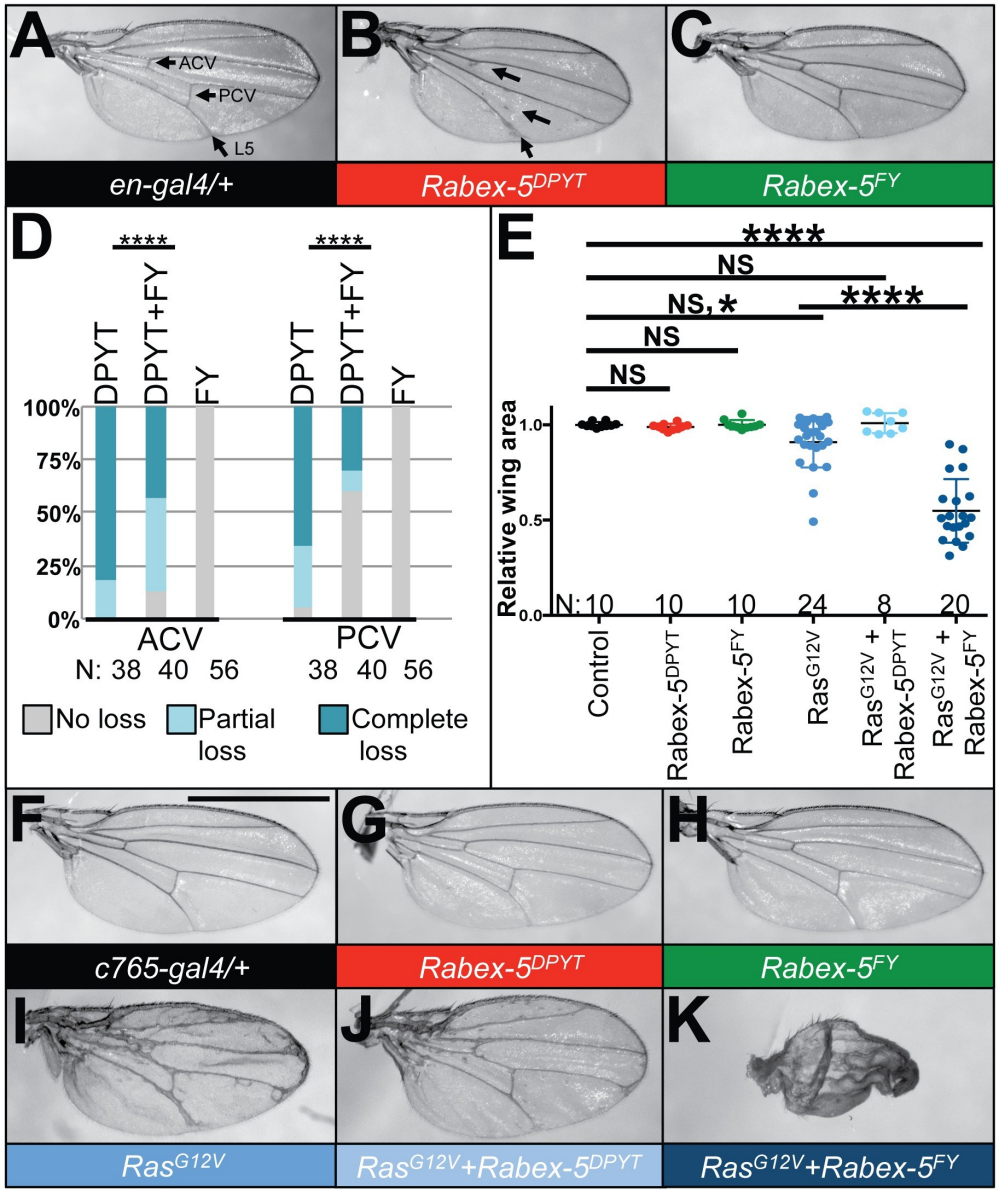
Rabex-5^DPYT^ suppresses oncogenic Ras phenotypes whereas Rabex-5 Rabex-5^FY^ enhances oncogenic Ras phenotypes in the wing. (A) Control *en-gal4/+* wing (genotype *w*^*1118*^*; en-gal4/+*). Arrows indicate the anterior crossvein (ACV), posterior crossvein (PCV) and L5. (B) Expressing Rabex-5^DPYT^ with *en-gal4* (genotype *w*^*1118*^*; en-gal4/UAS Rabex-5*^*DPYT*^). Arrows indicate loss of ACV and PCV, and partial loss of L5 where it meets the wing margin. (C) Expressing Rabex-5^FY^ with *en-gal4* (genotype *w*^*1118*^*; en-gal4/+; UAS Rabex-5*^*FY*^*/+*). (D) Graph quantifying partial and complete wing vein loss phenotypes for the ACV, PCV for Rabex-5^DPYT^, Rabex-5^FY^, and co-expressing Rabex-5^DPYT^ and Rabex-5^FY^ (genotypes*; en-gal4/UAS Rabex-5*^*DPYT*^, *w*^*1118*^*; en-gal4/+; UAS Rabex-5*^*FY*^*/+*, and *w*^*1118*^*; en-gal4/UAS Rabex-5*^*DPYT*^*; UAS Rabex-5*^*FY*^*/+*, respectively). Co-expressing Rabex-5^FY^ statistically significantly suppresses the PCV and ACV loss phenotypes of expressing Rabex-5^DPYT^. ****indicates p<0.0001 in chi square statistical tests. (E) Graph showing relative wing area for wings in F-K. One way ordinary ANOVA correcting for multiple comparisons using the Tukey test indicates that expressing Rabex-5^DPYT^ or Rabex-5^FY^ using *c765-gal4* does not change wing size (NS, P<0.9999). Expressing Ras^G12V^ reduced wing size in many wings leading to an average reduction of just over 9% compared to *c765-gal4/+* wings (NS,* indicates P = 0.2770 in Anova with multiple comparisons, P = 0.04 for unpaired T test). Expressing Rabex-5^DPYT^ suppressed the reduced wing size of expressing Ras^G12V^ to a size not significantly different than *c765-gal4/+* controls (NS, P>0.9999). Expressing Rabex-5^FY^ enhanced the reduced wing size of expressing Ras^G12V^ to a size statistically significantly different than *c765-gal4/+* controls (****, P<0.0001) and statistically significantly different than expressing Ras^G12V^ (****, P<0.0001). Given the increased variability in wing size for Ras^G12V^ and for Ras^G12V^ co-expressed with Rabex-5^FY^, double the number of wings were mounted to be more representative. (F) *c765-gal4/+* wing (genotype *w*^*1118*^*; c765-gal4/+*). Scale bar indicates 1mm and applies to wings in F-K. (G) Expressing Rabex-5^DPYT^ with *c765-gal4* (genotype *w*^*1118*^*; UAS Rabex-5*^*DPYT*^*/+; c765-gal4/+*). (H) Expressing Rabex-5^FY^ with *c765-gal4* (genotype *w*^*1118*^*; UAS Rabex-5*^*FY*^*/+; c765-gal4/+*). (I) Expressing Ras^G12V^ with *c765-gal4* (genotype *w*^*1118*^*; UAS Ras*^*G12V*^*/+; c765-gal4/+*). Wings have substantial phenotypes including ectopic vein material, thickened veins, and reduced size in some wings. (J) Co-expressing Ras^G12V^ and Rabex-5^DPYT^ in the wing with *c765-gal4* (genotype *w*^*1118*^*; UAS Rabex-5*^*DPYT*^*/Ras*^*G12V*^*; c765-gal4/+*). There is a suppression of wing vein phenotypes. (D) Co-expressing Ras^G12V^ and Rabex-5^FY^ in the wing with *c765-gal4* (genotype *w*^*1118*^*; UAS Rabex-5*^*FY*^*/Ras*^*G12V*^*; c765-gal4/+*). There is an obvious reduction in size and enhancement of wing vein phenotypes; wings are folded/crumpled.

The behavior of Rabex-5^FY^ to enhance Notch gain-of-function phenotypes prompted us to reexamine the relationship of Rabex-5 to Ras. Rabex-5 E3 activity (intact in Rabex-5^WT^ and Rabex-5^DPYT^) promotes the mono- and di-ubiquitination of Ras *in vitro* [4–6], and we previously showed that Rabex-5^WT^ and Rabex-5^DPYT^ clearly suppressed oncogenic Ras phenotypes in the differentiating eye *in vivo* [4]. Our published data also showed that Rabex-5^FY^ enhanced oncogenic Ras phenotypes in the differentiating eye [4] although this was not explicitly discussed in our prior study. As we reported previously [4], expressing Rabex-5^DPYT^ in the posterior wing with *en-gal4* caused loss of wing veins (Fig 3B), primarily the anterior crossvein (ACV) and the PCV, compared to controls (Fig 3A) consistent with Ras inhibition. If the Rab5 GEF domain promotes Ras signaling, then co-expressing Rabex-5^FY^ should suppress the phenotypes caused by Rabex-5^DPYT^. Indeed, expressing Rabex-5^FY^ with *en-gal4* to a level with no obvious wing vein phenotype (Fig 3C) statistically significantly suppressed the Rabex-5^DPYT^ wing vein loss phenotype (quantified in Fig 3D). One interpretation of this data is that these two catalytic domains act antagonistically in terms of Ras biological outputs.

To rule out that co-expressing Rabex-5^FY^ simply titrated away a factor required for Rabex-5^DPYT^ to regulate Ras and also to establish if Rabex-5^FY^ could directly promote Ras signaling, we performed interactions between the domain-specific transgenes and activated Ras mutant Ras^G12V^ (Fig 3E–3K). Expressing Ras^G12V^ in the wing with *c765-gal4* resulted in extra wing vein material (Fig 3I) and reduced size in many wings (quantified in Fig 3E) compared to a control wing (Fig 3F). Rabex-5^DPYT^ expressed using *c765-gal4* caused no obvious phenotype (Fig 3G) but suppressed the wing vein phenotypes of Ras^G12V^ (Fig 3J) as seen previously [6], and eliminated wings of reduced size (quantified in Fig 3E). In contrast, Rabex-5^FY^ caused no visible phenotype (Fig 3H) but enhanced Ras^G12V^ phenotypes, causing increased vein abnormalities, crumpling, and statistically significantly decreased wing size (Fig 3K; quantified in Fig 3E). Rabex-5^DPYT^ suppression of Ras^G12V^ phenotypes is consistent with Rabex-5 E3 activity inhibiting Ras as reported for the eye and wing [4,6]. Rabex-5^FY^ enhancement of Ras^G12V^ phenotypes is consistent with Rabex-5 Rab5 GEF activity enhancing activated Ras phenotypes as seen in the eye [4]. These data are consistent with a model that the Rab5 GEF domain promotes or amplifies Ras signaling similar to its behavior with Notch signaling (Fig 2). Taken together, Figs 2 and 3 could be interpreted that the two Rabex-5 catalytic domains, the E3 domain and the Rab5 GEF domain, have opposing activities towards Ras and Notch signaling. Rabex-5 also has a C-terminal coiled-coil domain implicated in binding Rabaptin-5, a protein that enhances Rabex-5 Rab5 GEF activity [1,27–29]. Previous structural studies [30] showed that deleting the ubiquitin-binding domain of Rabex-5 enhanced its nucleotide exchange activity. This would be consistent with our genetic interaction data using domain-specific inactivating mutations (rather than deletions) in the E3 domain. Curiously, this study also highlighted a role for ubiquitin binding in enhancing Rabex-5’s nucleotide exchange activity. It would be interesting for future studies to explore if Rabaptin-5 is involved in the relationship between Rabex-5’s E3 domain and its Rab5 GEF domain and to further resolve the role of ubiquitin binding and E3 activity in directly affecting Rab5 GEF activity rather than only indirectly by converging on the same downstream targets.

### Rabex-5 transgenes interact with PTEN/PI3K signaling

**Fig 4:**
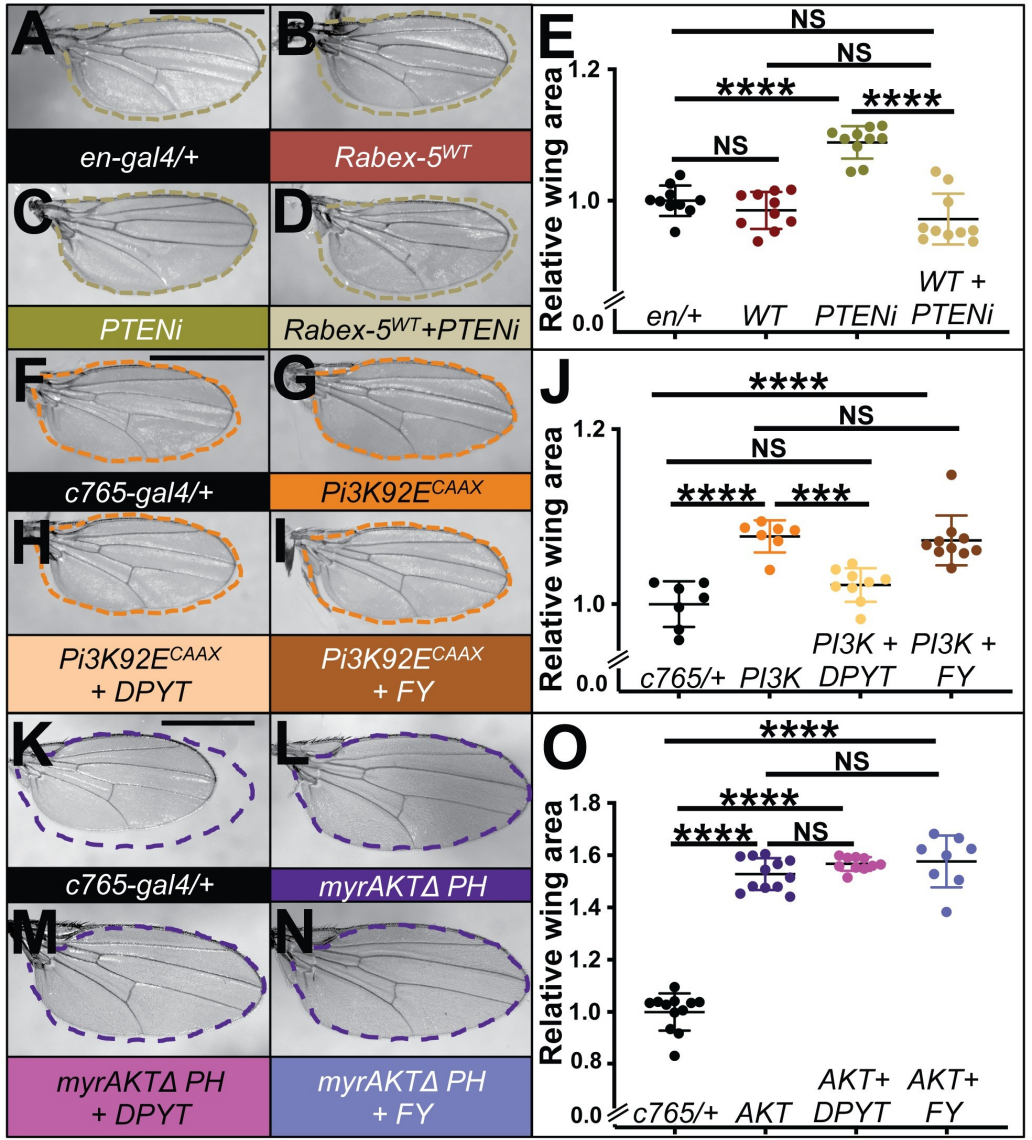
Rabex-5 interacts with PTEN/PI3K signaling in the wing. (A) Control *en-gal4* wing (genotype *w*^*1118*^*; en-gal4/+*). (B) Wing expressing Rabex-5^WT^ in the posterior wing with *en-gal4* (genotype *w*^*1118*^*; en-gal4*, *UAS Rabex-5*^*WT*^*/+*). (C) PTEN RNAi using allele *PTEN*^*JF01859*^ (PTENi) with *en-gal4* (genotype *w*^*1118*^*; en-gal4/+; UAS PTEN*^*JF01859*^*/+*). (D) Wing undergoing PTEN RNAi using allele *PTEN*^*JF01859*^ (PTENi) and expressing Rabex-5^WT^ in with *en-gal4* (genotype *w*^*1118*^*; en-gal4*, *UAS Rabex-5*^*WT*^*/+; UAS PTEN*^*JF01859*^*/+*). Dotted tracing of the wing in C is overlaid onto the wings in A, B, and D to highlight size differences. (E) Graph showing relative wing area of wings from A-D. Expressing Rabex-5^WT^ does not statistically significantly change wing size (NS, P = 0.6805) compared to control wings. PTEN RNAi using allele *PTEN*^*JF01859*^ (PTENi) statistically significantly increases wing size compared to control wings (****, P<0.0001). Expressing Rabex-5^WT^ suppresses wing overgrowth compared to PTEN RNAi alone (****, P<0.0001) to a size no longer statistically significantly different from *engal4/+* controls (NS, P = 0.1656) or *en>Rabex-5*^*WT*^ controls (NS, P = .7487). Overgrowth caused by distinct RNAi line *PTEN*^*HMS00044*^ is also suppressed by Rabex-5^WT^; data appears in S1 File. (F) Control *c765-gal4/+* wing (genotype *w*^*1118*^*; c765-gal4/+*). (G) Expressing Pi3K92E^CAAX^ using *c765-gal4* (genotype *UAS Pi3K92E*.*CAAX*, *y*^*1*^, *w*^*1118*^*; c765-gal4/+*). (H) Co-expressing Rabex-5^DPYT^ and Pi3K92E^CAAX^ using *c765-gal4* (genotype *UAS Pi3K92E*.*CAAX*, *y*^*1*^, *w*^*1118*^*; UAS Rabex-5*^*DPYT*^*/+; c765-gal4/+*). (I) Co-expressing Rabex-5^FY^ and Pi3K92E^CAAX^ using *c765-gal4* (genotype *UAS Pi3K92E*.*CAAX*, *y*^*1*^, *w*^*1118*^*; UAS Rabex-5*^*FY*^*/+; c765-gal4/+*). Dotted tracing of the wing in (G) is overlaid onto the wings in F, H, and I to highlight size differences. (J) Graphs showing relative wing area of wings in F-I). Expressing Pi3K92E^CAAX^ using *c765-gal4* statistically significantly increased wing size compared to controls (****, P<0.0001). Co-expressing Rabex-5^DPYT^ with Pi3K92E^CAAX^ suppresses wing overgrowth compared to Pi3K92E^CAAX^ expression alone (***, P = 0.0004) to a size no longer significantly different to control *c765-gal4/+* wings (NS, P = .2869). In multiple trials, co-expressing Rabex-5^FY^ concurrent to Pi3K92E^CAAX^ does not significantly change overgrowth compared to Pi3K92E^CAAX^ expression alone (NS, P = 0.9802). In contrast to (J), in 2 trials where there is a greater baseline of overgrowth, co-expressing Rabex-5^FY^ concurrent to Pi3K92E^CAAX^ significantly suppresses overgrowth compared to Pi3K92E^CAAX^ alone (****, P<0.0001), although this is still significantly overgrown compared to controls (****, P<0.0001) Data appears in S1 File. (K) Control *c765-gal4/+* wing (genotype *w*^*1118*^*; c765-gal4/+*). (L) Expressing myr-AKT-DeltaPH using *c765-gal4* (genotype *w*^*1118*^*; c765-gal4/UAS myr-AKT-DeltaPH*). (M) Co-expressing Rabex-5^DPYT^ and myr-AKT-DeltaPH using *c765-gal4* (genotype *w*^*1118*^*; UAS Rabex-5*^*DPYT*^*/+; c765-gal4/UAS myr-AKT-DeltaPH*). (N) Co-expressing Rabex-5^FY^ and myr-AKT-DeltaPH using *c765-gal4* (genotype *w*^*1118*^*; UAS Rabex-5*^*FY*^*/+; c765-gal4/UAS myr-AKT-DeltaPH*). Dotted tracing of the wing in (L) is overlaid onto the wings in K, M, and N to highlight size differences or lack of difference. (O) Graphs showing relative wing area of wings in K-N. Expressing myr-AKT-DeltaPH using *c765-gal4* statistically significantly increased wing size compared to controls (****, P<0.0001). Co-expressing Rabex-5^DPYT^ with myr-AKT-DeltaPH or Rabex-5^FY^ does not significantly change overgrowth compared to myr-AKT-DeltaPH expression alone (NS, P = 0.5338 for Rabex-5^DPYT^, P = 0.4015 for Rabex-5^FY^); wings are still statistically significantly increased for both compared to control wings (****P<0.0001). Statistical analysis in E, J, and O used ordinary one-way ANOVA with Tukey’s multiple comparison test. Scale bars in A, F, and K indicate 1mm and apply to wings in A-D, F-I, and K-N respectively.

The ability of Rabex-5 to regulate both Ras and Notch raised the question of whether Rabex-5 function is restricted to Ras and Notch regulation. We began screening for Rabex-5^WT^ interactions with other signaling cascades using *en-gal4* which, as noted, drives strong expression of transgenes in the posterior wing. We uncovered an interaction between over-expressing Rabex-5^WT^ and loss of *PTEN* (Phosphatase and tensin homolog). RNAi to PTEN in the posterior wing using *en-gal4* caused wing overgrowth (Fig 4C, quantified in Fig 4E) compared to controls (Fig 4A). Rabex-5 co-expression to a level that caused wing vein phenotypes but did not change wing size (Fig 4B) suppressed this overgrowth for distinct PTEN RNAi lines *PTEN*^*JF01859*^ and *PTEN*^*HMS00044*^ (shown for *PTEN*^*JF01859*^ Fig 4D, quantified in Fig 4E; data for a trial with *PTEN*^*HMS00044*^ is included in S1 File).

PTEN is a phosphatase in the insulin signaling cascade that antagonizes PI3K by removing the PI3K-placed phosphorylation on PIP3. Therefore, we next tested Rabex-5 regulation of PI3K using the catalytic subunit p110, referred to as *Pi3K92E* in *Drosophila*. Ras can promote PI3K activity. Therefore, we utilized a transgene encoding Pi3K92E with a farnesylation signal, *UAS Pi3K92E*^*CAAX*^ [31], which should not rely on Ras for activation. If Rabex-5 regulates insulin signaling via its regulation of PI3K by Ras, we would predict Rabex-5 would not be able to inhibit the constitutively active Pi3K92E^CAAX^. If Rabex-5 regulates insulin signaling at the step of PI3K, on a Ras-independent PI3K regulator, or downstream of PI3K, we would expect interactions similar to those seen for RNAi of PTEN. To address the requirement for each of the catalytic domains, rather than use *en-gal4* which produced strong phenotypes for Rabex-5^DPYT^ [4] (Fig 3B) that could interfere with wing size interpretations, we instead expressed the Rabex-5 domain-specific mutant transgenes using *c765-gal4* because we had shown that this led to no visible phenotypes by (Figs 2D, 2E, 3E, 3G and 3H). Expressing Pi3K92E^CAAX^ with *c765*-*gal4* led to ~10–20% overgrowth (Fig 4G, quantified in Fig 4J) compared to controls (Fig 4F). Co-expressing Rabex-5^DPYT^ completely suppressed the overgrowth, returning wings to a size no longer statistically different from control wings (Fig 4H, quantified in Fig 4J). This demonstrates that Rabex-5 does not require its Rab5 GEF activity to modify PI3K size phenotypes and is consistent with a model that its E3 domain can inhibit PI3K-mediated overgrowth independent of Ras. In contrast to the activity of Rabex-5^FY^ to enhance Notch and Ras over-expression phenotypes, co-expressing Rabex-5^FY^ did not enhance Pi3K92E^CAAX^-mediated overgrowth (Fig 4I quantified in Fig 4J). In several experiments, co-expressing Rabex-5^FY^ had no effect on Pi3K92E^CAAX^-mediated overgrowth (quantified in Fig 4J); curiously, in two trials, co-expressing Rabex-5^FY^ suppressed some overgrowth in a limited fashion (quantified in S1 File). This suppression was not as extensive as that by Rabex-5^DPYT^, and the overgrowth was still significantly statistically different from controls. Because the behavior of Rabex-5^FY^ to suppress overgrowth was inconsistent, we can conclude that Rabex-5^FY^ does not enhance Pi3K92E^CAAX^ overgrowth phenotypes; however, we cannot conclude or rule out a role for Rabex-5^FY^ to suppress Pi3K92E^CAAX^ overgrowth phenotypes.

PI3K phosphorylates PIP2 to create PIP3 which then activates AKT. We utilized a transgene encoding a myristoylated form of AKT with the pleckstrin homology (PH) domain deleted, *UAS myr-AKT-DeltaPH*, which localizes to the membrane and is constitutively active. If Rabex-5 regulates insulin signaling at the step of AKT, on a PI3K-independent AKT regulator, or downstream of AKT, we would expect interactions similar to those seen for RNAi of PTEN and expression of PI3K92E^CAAX^. In contrast, under conditions in which driving expression of myr-AKT-DeltaPH using *c765-gal4* led to ~60% overgrowth (Fig 4L, quantified in Fig 4O) compared to a control wing (Fig 4K), both Rabex-5^DPYT^ (Fig 4M) and Rabex-5^FY^ (Fig 4N) failed to modify the myr-AKT-DeltaPH phenotypes. Therefore, we speculate that Rabex-5 inhibits insulin signaling at the step of PTEN and PI3K or on a PTEN or PI3K regulator, not further downstream. However, because this AKT transgene lacked the PH domain, we cannot rule out the possibility that Rabex-5 requires the PH domain to inhibit AKT. Importantly, the failure of Rabex-5^DPYT^ to modify myr-AKT-DeltaPH-mediated overgrowth together with our previous finding that Rabex-5 cannot target forms of Ras that cannot be phosphorylated at Y4 [6] reinforces that Rabex-5 inhibition of downstream activities is selective.

A role for Rabex-5 to inhibit signaling through Ras, Notch, and PI3K is consistent with our previous work [4] and other work in *Drosophila* that reported overgrowth phenotypes upon loss of Rabex-5 [32]. Overgrowth upon Rabex-5 loss is consistent with a tumor suppressor role for Rabex-5; indeed, SAGE and genomic studies show deletion of Rabex-5 in several cancers including leukemias, lymphomas, and pancreatic cancer [33–36]. In contrast, many cancer studies show amplification of Rabex-5, including reports that Rabex-5 is an oncogene and is associated with poor prognosis in gastric cancers [37,38], colorectal cancer [39], lung cancer [40,41], breast cancer [42], and prostate cancer [43].

**Fig 5:**
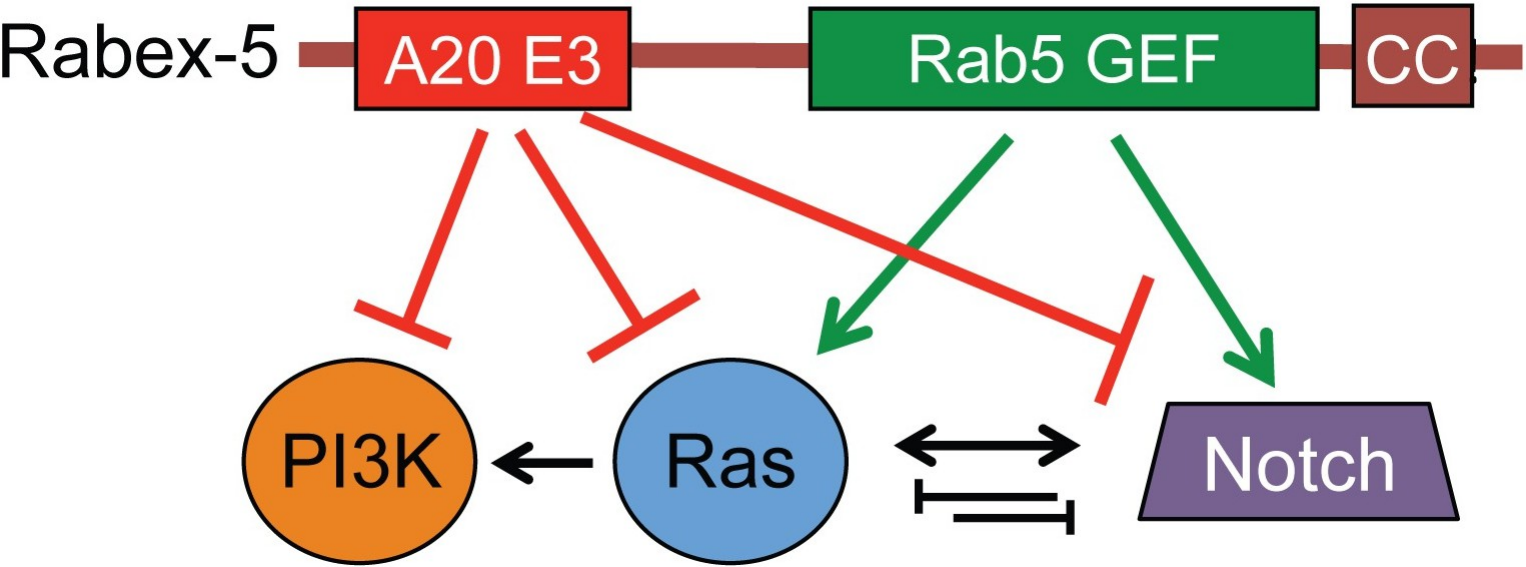
Proposed model for Rabex-5 regulation of Ras, Notch, and PI3K. We propose that the Rabex-5 E3 domain inhibits Ras, Notch, and PI3K signaling and the Rabex-5 Rab5 GEF domain promotes Ras and Notch signaling. Previous work [4,5] indicated a direct role for Rabex-5 to inhibit Ras by ubiquitination, but the directness and target of Rabex-5 E3 activity in Notch and PI3K signaling has not been identified. Given the role of Ras in activating PI3K and the complicated and context-dependent relationship between Ras and Notch, Rabex-5 domain-specific activity for these signaling cascades adds another layer of regulation to fine-tune signaling outputs during development and could have tremendous relevance to disease states that rely on amplified Ras, Notch, or PI3K.

A role for Rabex-5 as an oncogene and a tumor suppressor presents a paradox. We propose that the balance of the two catalytic domains of Rabex-5 toward downstream signaling networks works to fine-tune their outputs to establish proper developmental patterning (model, Fig 5) and to define Rabex-5 ability to act as an oncogene or a tumor suppressor. We speculate that the Rabex-5 E3 domain acts as a tumor suppressor by inhibiting signaling through Ras, Notch, and PI3K signaling, and that the Rab5 GEF domain acts as an oncogene by enhancing signaling through Ras and Notch. This may be particularly relevant to specific cancer types that amplify Rabex-5 and one or more of these oncogenes. To test this model and elucidate this mechanistically, it will be important for subsequent efforts to address how each domain affects direct transcriptional and post-translational targets of each of these signaling cascades and how Rabex-5 inhibitors and activators modulate these effects.

Ras and Notch signaling can act antagonistically or synergistically to each other depending on context [44]. PI3K can be activated by Ras or independently of Ras. Each of these signaling cascades plays a number of important roles during development and has been implicated in a variety of diseases. The ability of Rabex-5^DPYT^ to suppress phenotypes caused by dysregulating these important signaling cascades combined with the ability of Rabex-5^FY^ to enhance the phenotypes of activated Ras and Notch may implicate Rabex-5 in an important regulatory role to fine-tune the eventual biological outputs in development and which could be important in disease contexts.

Previous work from our group and others [4,5] established that the Rabex-5 E3 domain promotes ubiquitination of Ras itself requiring a tyrosine-based motif [6]; it will be important for future efforts to identify the ubiquitination targets of Rabex-5 E3 activity in Notch and PI3K networks and establish if this targeting requires the same tyrosine signal. Rabex-5 Rab5 GEF activity has been associated with regulation of endosomal trafficking [1]. It will be important for future efforts to establish the direct impacts of Rabex-5 Rab5 GEF activity on the trafficking of Ras and Notch signaling components to elucidate mechanistically how this activity enhances their oncogenic signaling.

## Materials and methods

### Rigor and reproducibility

The reported work represents reproducible experiments that reflect a minimum of three well-controlled, independent trials. To avoid observer bias, trials for the same experiment were conducted by at least two different lab members independently including high school and undergraduate students who examined samples without knowledge of anticipated outcomes. For phenotypes that are subjective (not quantifiable), independent lab members scored progeny blind to avoid bias.

### Statistical analysis

Chi-square analysis and Fisher’s exact tests were applied as appropriate using contingency tables for categorical scoring in GraphPad Prism compare fly wings with specific phenotypes in Figs 1K, 1L, 2F, 2G and 3D. Wings in Figs 3 and 4 were measured using ImageJ software. Wing size comparisons were analyzed using GraphPad Prism software one way ANOVA analysis for multiple comparisons using the Tukey test in Figs 3E, 4E, 4J and 4O. Wing size comparison between *c765-gal4/+ and UAS Ras*^*G12V*^*/+; c765-gal4/+* wings cited in the Fig 3 legend was also calculated using GraphPad Prism unpaired T-test. P values for statistical analysis are listed in S1 File.

### Drosophila

Gal4 drivers were obtained from the Bloomington Drosophila Stock Center or other labs in the Drosophila community. *PTEN* RNAi lines (P{TRiP.JF01859}attP2, BL-25841, and P{TRiP.HMS00044}attP2, BL-33643), *UAS Pi3K92E*^*CAAX*^ (UAS Pi3K92E.CAAX, BL-8294) and *UAS myr-AKT-DeltaPH* (BL-80935) were from the Bloomington Stock center. UAS Rabex-5^WT,^ UAS Rabex-5^DPYT^, and UAS Rabex-5^FY^ were characterized in our previous study [4]. A full list of strains and sources in Table 1. Crosses were performed on standard *Drosophila* medium. Specific experiments shown in figures were performed at the temperatures indicated in the figure legends. Crosses were performed and reproduced at both 21°C and 25°C resulting in comparable phenomena with the following exceptions: experiments in Fig 2F were performed and reproduced only at 25°C; experiments in Fig 2G were performed and reproduced only at 21°C; driving Ras^G12V^ at 25°C is lethal and driving myr-AKT-DeltaPH increases lethality so experiments in Fig 3E and 3I–3K were performed only at 21°C, and experiments in 4K-O were only reproduced with full datasets of three trials at 21°C. Female wing images and data are shown. All wings within individual experiments were photographed at the same magnification to allow for relative size comparisons between images. Raw wing images were converted to grayscale and cropped in Adobe Photoshop. The same degree of resizing and cropping were applied in parallel to all images from the same individual experiments to allow for comparisons to be made between figure panels. Brightness and contrast of wing images were adjusted in Adobe Photoshop to maximize clarity; adjustments were applied to the entire images. Wings were measured in ImageJ for graphs and analysis shown in Figs 3E, 4E, 4J and 4O. Genotypes are summarized below.

### Genotypes of flies in images or graphs

*w*^*1118*^*; FRT80B/+* (Fig 1A)

*N*^*55e11*^*/w*^*1118*^*; FRT80B/+* (Fig 1B, 1B’, 1C and 1C’; Graph in Fig 1K)

*N*^*55e11*^*/w*^*1118*^*; FRT80B Rabex-5*^*ex42*^*/+* (Fig 1G–1H’; Graph in Fig 1K)

*DpN/+; FRT80B/+* (Fig 1D, 1D’, 1E and 1E’; Graph in Fig 1L)

*w*^*1118*^*; FRT80B Rabex-5*^*ex42*^*/+* (Fig 1F)

*DpN/+; FRT80B Rabex-5*^*ex42*^*/+* (Fig 1I–1J’; Graph in Fig 1L)

*w*^*1118*^*; c765-gal4/+* (Fig 2A, Graph in Fig 3E, 3F, 4F and 4K; Graphs in Fig 4J and 4O)

*w*^*1118*^*; UAS Rabex-5*^*WT*^*/+; c765-gal4/+* (Fig 2C)

*w*^*1118*^*; UAS Rabex-5*^*DPYT*^*/+; c765-gal4/+* (Fig 2D, Graph in Fig 3E and 3G)

*w*^*1118*^*; UAS Rabex-5*^*FY*^*/+; c765-gal4/+* (Fig 2E, Graph in Fig 3E and 3H)

*N*^*55e11*^*/w*^*1118*^*; c765-gal4/+* (Graph in Fig 2F and 2F’)

*N*^*55e11*^*/w*^*1118*^*; UAS Rabex-5*^*WT*^*/+; c765-gal4/+* (Graph in Fig 2F)

*N*^*55e11*^*/w*^*1118*^*; UAS Rabex-5*^*DPYT*^*/+; c765-gal4/+* (Graph in Fig 2F–2F”)

*N*^*55e11*^*/w*^*1118*^*; UAS Rabex-5*^*FY*^*/+; c765-gal4/+* (Graph in Fig 2F)

*w*^*1118*^*; DpN/+; c765-gal4/+* (Graph in 2G)

*w*^*1118*^*; UAS Rabex-5*^*WT*^*/DpN; c765-gal4/+* (Graph in 2G)

*w*^*1118*^*; UAS Rabex-5*^*DPYT*^*/DpN; c765-gal4/+* (Graph in 2G)

*w*^*1118*^*; UAS Rabex-5*^*FY*^*/DpN; c765-gal4/+* (Graph in 2G)

*w*^*1118*^*; en-gal4/+* (Fig 3A and 4A, Graph in 4E)

*w*^*1118*^*; en-gal4/UAS Rabex-5*^*DPYT*^ (Fig 3B, Graph in 3D)

*w*^*1118*^*; en-gal4/+; UAS Rabex-5*^*FY*^*/+* (Fig 3C, Graph in 3D)

*w*^*1118*^*; en-gal4/UAS Rabex-5*^*DPYT*^*; UAS Rabex-5*^*FY*^*/+* (Graph in 3D)

*w*^*1118*^*; UAS Ras*^*G12V*^*/+; c765-gal4/+* (Graph in Fig 3E and 3I)

*w*^*1118*^*; UAS Rabex-5*^*DPYT*^*/Ras*^*G12V*^*; c765-gal4/+* (Graph in Fig 3E and 3J)

*w*^*1118*^*; UAS Rabex-5*^*FY*^*/Ras*^*G12V*^*; c765-gal4/+* (Graph in Fig 3E and 3K)

*w*^*1118*^*; en-gal4*, *UAS Rabex-5*^*WT*^*/+* (Fig 4B, Graph in 4E)

*w*^*1118*^*; en-gal4/+; UAS PTEN*^*JF01859*^*/+* (Fig 4C, Graph in 4E)

*w*^*1118*^*; en-gal4*, *UAS Rabex-5*^*WT*^*/+; UAS PTEN*^*JF01859*^*/+* (Fig 4D, Graph in 4E)

*UAS Pi3K92E*.*CAAX*, *y*^*1*^, *w*^*1118*^*; c765-gal4/+* (Fig 4G; Graphs in Fig 4J and 4J’)

*UAS Pi3K92E*.*CAAX*, *y*^*1*^, *w*^*1118*^*; UAS Rabex-5*^*DPYT*^*/+; c765-gal4/+* (Fig 4H; Graphs in Fig 4J and 4J’)

*UAS Pi3K92E*.*CAAX*, *y*^*1*^, *w*^*1118*^*; UAS Rabex-5*^*FY*^*/+; c765-gal4/+* (Fig 4I; Graphs in Fig 4J and 4J’)

*w*^*1118*^*; c765-gal4/UAS myr-AKT-DeltaPH* (Fig 4L; Graph in Fig 4O)

*w*^*1118*^*; UAS Rabex-5*^*DPYT*^*/+; c765-gal4/UAS myr-AKT-DeltaPH* (Fig 4M; Graph in Fig 4O)

*w*^*1118*^*; UAS Rabex-5*^*FY*^*/+; c765-gal4/UAS myr-AKT-DeltaPH* (Fig 4N; Graph in Fig 4O)

**Table 1:** Table of reagents used with corresponding identifiers.

Drosophila Strains		
Strain	Source	Identifier
*w* ^*1118*^	The fly community and Bloomington Drosophila Stock Center (BDSC)	BL-3605, BL-5905 and others RRID:BDSC_3605, RRID:BDSC_5905
*FRT80B*	BDSC	Can be obtained from BDSC, 1988 RRID:BDSC_1988
*N* ^*55e11*^	Provided by M. Mlodzik and U. Weber.	
*DpN*	Provided by the NYC fly community and used in our previous study Reimels and Pfleger, 2015 [7]	
*FRT80B Rabex-5* ^*ex42*^	Yan et al., 2010 [4]	
*en-gal4*	Provided by the Hariharan lab	
*UAS Rabex-5* ^*WT*^	Yan et al., 2010 [4]	
*UAS Rabex-5* ^*DPYT*^	Yan et al., 2010 [4]	
*UAS Rabex-5* ^*FY*^	Yan et al., 2010 [4]	
*w; c765-gal4*	BDSC, NYC fly community	Can be obtained from BDSC, BL-36523 RRID:BDSC_36523
*UAS Flag-His6-Ras* ^*G12V*^	Washington et al., 2020 [6]	
*UAS PTEN* ^*JF01859*^	BDSC	Can be obtained from BDSC, BL-25841 RRID:BDSC_25841
*UAS PTEN*	BDSC	Can be obtained from BDSC, 33643 RRID:NDSC_33643
*UAS Pi3K92E* ^*CAAX*^	BDSC	Can be obtained from BDSC, 8294 RRID:NDSC_8294
*UAS myr-AKT-DeltaPH*	BDSC	Can be obtained from BDSC, 80935 RRID:NDSC_80935
**Software**		
ImageJ		https://imagej.nih.gov/ij/
Adobe Photoshop		https://www.adobe.com/products/photoshop.html
GraphPad Prism		https://www.graphpad.com/scientific-software/prism/
Microsoft Excel		https://www.microsoft.com/Microsoft/Excel/

## Supporting information

S1 File This excel file contains sheets corresponding to the data presented in Figs 1–4. Data from each figure is compiled in a sheet of the same name. P values for all analysis in each figure are summarized in the sheet subsequent to the corresponding figure.
(XLSX)

## Abbreviations

ACV: Anterior crossvein
PCV: Posterior crossvein
